# Effect of Tempering Temperature on Microstructural Evolution and Mechanical Properties of Cr-Ni-Mo-V Steel for Pressure Vessel Applications

**DOI:** 10.3390/ma19091679

**Published:** 2026-04-22

**Authors:** Enpu Liang, Xiaodong Liang, Yong Yang, Wenchao Yu, Le Xu, Maoqiu Wang, Jie Shi

**Affiliations:** 1 Research Institute for Special Steels, Central Iron and Steel Research Institute Co., Ltd. (CISRI), Beijing 100081, China; liangenpu24@163.com (E.L.);; 2 Fushun Special Steel Co., Ltd., Fushun 113000, China; 3 Chongqing Changan Wangjiang Industrial Group Co., Ltd., Chongqing 404135, China

**Keywords:** Cr-Ni-Mo-V steel, tempering temperature, microstructure, mechanical properties, carbides

## Abstract

This study investigates the effects of tempering temperature on the microstructural evolution and mechanical properties of Cr-Ni-Mo-V steel designed for pressure vessel applications. The microstructure was characterized via scanning electron microscopy (SEM), transmission electron microscopy (TEM, Thermo Fisher Talos F200X), electron backscatter diffraction (EBSD), and physicochemical phase analysis. Mechanical performance was evaluated through tensile and impact tests, followed by a detailed discussion of the underlying strengthening mechanisms. The results demonstrate that the microstructure after tempering is fully tempered martensite. Samples tempered between 425 °C and 525 °C exhibit significant tempering resistance, maintaining a tensile strength of approximately 1300 MPa. This is primarily attributed to the synergistic effect of dislocation strengthening and the precipitation of MC-type carbides. As the tempering temperature increases to 625 °C, the dislocation density decreases sharply from 3.71 × 10^11^ cm^−2^ to 1.18 × 10^11^ cm^−2^, leading to a decline in strength. Concurrently, the impact energy increases significantly from 71 J to 132 J. The improvement in toughness is mainly attributed to the significant elevation of the crack initiation threshold, which is dominated by the reduction in matrix dislocation density, the coarsening and spheroidization of carbides, and the alleviation of local stress concentration. The relative proportion of high-angle grain boundaries (HAGBs, misorientation > 15°) increases from 51.9% to 57.7% during tempering, which is a result of the massive elimination of low-angle grain boundaries rather than an increase in the absolute length per unit area of HAGBs.

## 1. Introduction

With the continuous development of modern industry towards large-scale, high-efficiency, long-service-life and high-safety operation, the performance requirements for key load-bearing structural components in extreme service environments have become increasingly stringent [1,2]. In the fields of thick-walled pressure vessels, heavy hydropower equipment, large-scale engineering machinery and steam turbine rotors, the core requirement for structural materials is to achieve an optimal combination of high strength, excellent plasticity and superior toughness, which directly determines the structural integrity, long-term service reliability and operational safety of the equipment [3,4]. Against this background, Cr-Ni-Mo-V martensitic steel has become the preferred material for manufacturing large forgings for the above-mentioned equipment, especially thick-walled pressure vessels that bear long-term high-pressure and alternating loads. This is attributed to its excellent comprehensive mechanical properties, outstanding hardenability, good tempering resistance, and high controllability of microstructure and properties via heat treatment processes [5,6].

Quenching and tempering (Q&T), as the most critical heat treatment process for tailoring the final microstructure and service properties of Cr-Ni-Mo-V steel, has been the core focus of existing research in this field [7]. The quenching process determines the hierarchical substructure of the initial lath martensite, while the subsequent tempering process dominates the decomposition of metastable supersaturated martensite, the recovery and annihilation of dislocations, the evolution of grain boundary characteristics, and the precipitation and coarsening behavior of secondary carbide phases [8,9]. By precisely controlling the key parameters of the Q&T process, especially the tempering temperature and holding time, the multi-scale microstructure of the steel can be effectively regulated, and then the optimal matching of strength, plasticity and toughness can be achieved [10]. At present, a large number of studies have systematically explored the influence of tempering temperature on the microstructure evolution and mechanical properties of Cr-Ni-Mo-V system steels, and obtained a series of quantitative conclusions. In terms of the influence of tempering temperature on the macroscopic mechanical properties of Cr-Ni-Mo-V steel, existing studies have generally confirmed that the strength of the steel decreases while the toughness increases with increasing tempering temperature, and given quantitative characterization results for different component systems [11,12,13,14,15,16,17,18,19,20,21,22,23,24,25]. Zhao carried out a systematic study on Cr-Ni-Mo-V rotor steel with a chemical composition of 0.26 wt.% C, 1.52 wt.% Cr, 3.52 wt.% Ni, 0.48 wt.% Mo and 0.20 wt.% V, and the results showed that when the tempering temperature increased from 500 °C to 660 °C, the tensile strength of the steel decreased from 1350 MPa to 980 MPa, while the room temperature impact energy increased from 34 J to 210 J, showing a significant trade-off relationship between strength and toughness with the change in tempering temperature [6]. Yan studied the effect of tempering temperature on Cr-Mo-V-Ni steel, and found that when the tempering temperature increased from 200 °C to 600 °C, the tensile strength of the steel decreased from 1900 MPa to 1100 MPa, and the low-temperature impact energy at −40 °C increased from 15 J to 50 J, further verifying the general law of the effect of tempering temperature on the mechanical properties of this system steel [16].

In terms of the effect of tempering temperature on the multi-scale microstructure evolution of Cr-Ni-Mo-V steel, existing studies have carried out in-depth discussions from three dimensions, namely, dislocation density evolution, grain boundary characteristic change and carbide precipitation behavior, and formed clear quantitative conclusions. For the evolution of dislocation density, Zhou et al. [13] found that for Cr-Mo-V steel, when the tempering temperature increased from 200 °C to 650 °C, the dislocation density in the matrix decreased from 4.2 × 10^15^ m^−2^ to 0.8 × 10^15^ m^−2^, and the contribution of dislocation strengthening to the yield strength of the steel decreased by more than 60%, which was confirmed as the core reason for the strength decline of the steel during tempering. The researchers further pointed out that the high dislocation density formed during martensitic transformation is the core source of the high strength of low-alloy martensitic steel, and the recovery, rearrangement and annihilation of dislocations during tempering directly determine the attenuation law of the strength of the steel. For the evolution of grain boundary characteristics [2], Jiang carried out a quantitative study on 42CrMo4V steel, and the results showed that when the tempering temperature increased from 200 °C to 650 °C, the proportion of high-angle grain boundaries (HAGBs, misorientation > 15°) in the steel increased from 42% to 58%, and the corresponding low-temperature impact energy was nearly doubled, which confirmed that the increase in HAGB proportion is the key factor to improve the toughness of the steel [26]. Zhao also found that increasing the proportion of HAGBs can effectively hinder the propagation of cracks in the steel, consume more fracture energy, and be the core microstructural factor to coordinate the strength-toughness balance of martensitic steel [11]. For the precipitation behavior of carbides, existing studies have clearly established the typical precipitation sequence of carbides in low-alloy martensitic steels during tempering, namely, MC + M_3_C → MC → MC + M_2_C + M_7_C_3_ → MC + M_7_C_3_ + M_23_C_6_ [25,26,27], and clarified the different effects of different types of carbides on the mechanical properties of the steel. Among them, M_7_C_3_ phase has insufficient strengthening effect at high temperature, M_3_C phase nucleated at the interface is easy to cause steel embrittlement, and although M_2_C phase can improve strength and toughness at the same time, its coarsened form will damage the impact resistance of the steel [28]. In contrast, MC-type carbides have high thermal stability in a wide temperature range, which can effectively improve the strength of the steel without causing significant loss of toughness [29,30,31]. Zheng pointed out that nano-scale MC carbides can increase the yield strength of the steel by more than 200 MPa while maintaining the impact energy no less than 80 J, which is the core secondary phase to achieve the strength–toughness balance of Cr-Ni-Mo-V steel [28]. Additionally, the precipitation of nano-scale MC-type alloy carbides during medium- to high-temperature tempering induces a significant secondary hardening effect, which is the core mechanism enabling low-alloy martensitic steels to maintain high strength and excellent tempering resistance. This effect has been systematically confirmed in numerous classical and modern studies, forming the fundamental theoretical basis for the composition design and heat treatment process optimization of Cr-Ni-Mo-V system martensitic steels [22,23].

Comprehensive analysis indicates that tempering temperature significantly influences the properties of Cr-Ni-Mo-V steel by regulating the evolution of the matrix and precipitates. Proper matching of composition design and tempering process parameters is crucial to meet the stringent requirements of high strength and excellent toughness for thick-walled pressure vessel steels. Based on the traditional Cr-Ni-Mo-V steel composition system, this study designed and fabricated a new medium-carbon high-nickel Cr-Ni-Mo-V steel through optimizing the content of key alloying elements, with composition optimization as the core driving force to achieve a superior balance of strength and toughness. Under fixed tempering time, a systematic experimental approach combined with multi-scale microstructural characterization methods was employed to quantitatively investigate the effects of tempering temperature on the microstructural evolution and mechanical properties of the experimental steel, the intrinsic correlation mechanisms between tempering temperature and key mechanical performance indicators such as strength, plasticity, and toughness were revealed. Ultimately, this study provides theoretical foundations and technical guidance for the synergistic optimization of alloy composition and heat treatment process parameters for pressure vessel steels in industrial applications.

## 2. Materials and Methods

### 2.1. Experimental Materials

**Figure 1:**
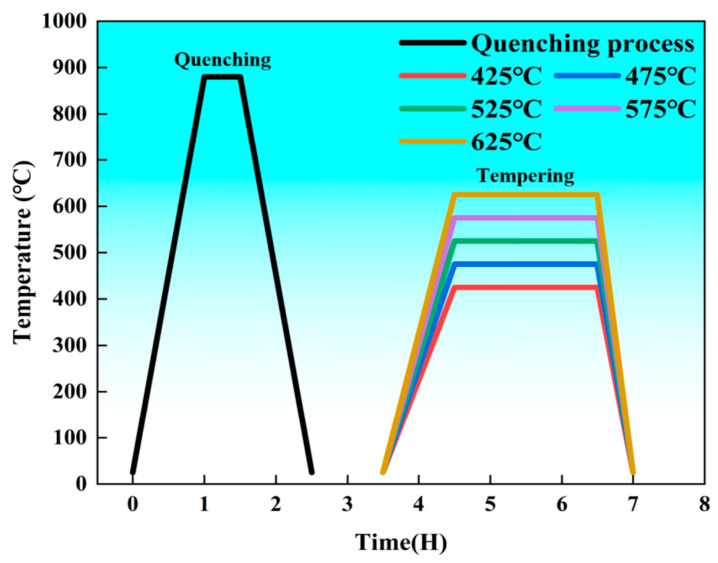
Schematic illustration of the heat treatment process for Cr-Ni-Mo-V steel.

The experimental Cr-Ni-Mo-V steel was fabricated in a 50 kg vacuum induction furnace under an argon-shielded atmosphere and subsequently cast into a copper mold. The chemical composition was characterized using optical emission spectroscopy, with the results summarized in Table 1. The ingot obtained by smelting was homogenized at 900 °C for 8 h to soften and anneal, aiming to eliminate the residual internal stress from ingot solidification, reduce the hardness of the as-cast structure, and improve the machinability and hot workability of the material. This treatment also stabilizes the matrix structure, providing a microstructural foundation for the subsequent hot working and final heat treatment. After being forged at 1200 °C, the ingot was processed to a total forging ratio of no less than 6:1. Hot working with large deformation can obviously break the as-cast structure and reduce macro-segregation, and finally obtain a round bar with Φ 16 mm× 1000 mm. Prior to Q&T, 25 mm sections were removed from both ends of each bar to eliminate potential effects of macro-segregation on mechanical performance. Blank specimens (Φ 16 mm × 75 mm) were machined from the remaining bars for the heat treatment regimen. All mechanical properties and microscopic characterization specimen are taken from 1/2 radius of the round bar, completely avoiding the central segregation zone and edge area; the gauge length section of the tensile sample and the V-shaped notch area of the impact sample were strictly controlled within the same annular zone at 1/2 radius to ensure the consistency of the sampling positions of all samples and minimize the impact of segregation on the test results. The specimens were initially heated from room temperature to an austenitizing temperature of 880 °C over a duration of 2 h, held for 0.5 h, and then oil-quenched to room temperature. Subsequently, the quenched specimens were tempered at 425 °C, 475 °C, 525 °C, 575 °C, and 625 °C, respectively. The tempering stage involved a 2 h heating ramp from room temperature to the target temperature, followed by a 2 h isothermal holding period and final water quenching [6]. The heat treatment profiles are schematically illustrated in Figure 1.

It should be noted that although forging and homogenization treatments significantly reduce macrosegregation, microsegregation of alloying elements, particularly strong carbide-forming elements such as V, Mo, and Cr, may still persist. This can lead to the presence of coarse, undissolved carbides in the quenched microstructure, which may not fully dissolve during the austenitizing process at 880 °C for 2 h. The presence of undissolved carbides will lead to local inhomogeneity of microstructure (including lath width, dislocation density, and tempering carbide precipitation behavior), while the mechanical properties and dislocation density results are averaged over a large sampled volume, and thus the overall impact on the macroscopic performance trends is negligible. However, this factor has been fully considered in the statistical error analysis of microstructure characterization and quantitative calculation of strengthening mechanisms.

### 2.2. Experimental Methods

**Figure 2:**
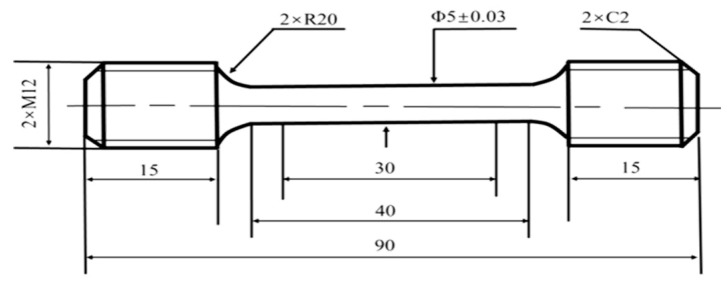
Schematic geometry and dimensions of the tensile specimens for Cr-Ni-Mo-V steel [6].

**Figure 3:**
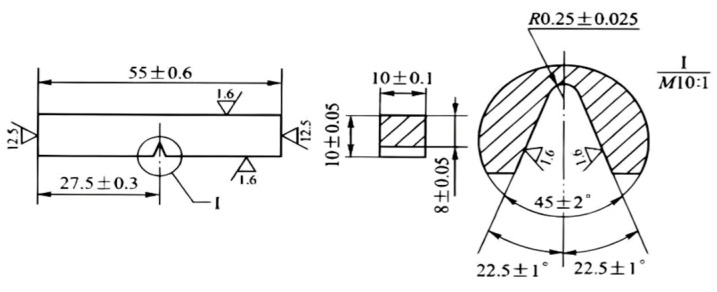
Geometry and dimensions of the standard impact specimen for Cr-Ni-Mo-V steel.

Room-temperature tensile tests and sample preparation were performed in accordance with the Chinese national standard GB/T 228.1-2021 [32]. The heat-treated blanks were machined into standard tensile specimens with a gauge diameter of 5 mm and a gauge length of 25 mm, as illustrated in Figure 2. Testing was conducted on a WE-300 universal testing machine (Jinan Touching Group Co., Ltd., Jinan, China) at a constant strain rate of 2.5 × 10^−4^ s^−1^ until fracture. To ensure statistical reliability, three specimens were tested for each heat treatment condition. Charpy V-notch impact tests and sample preparation at room temperature were conducted in accordance with the GB/T 229-2020 standard [33]. The heat-treated specimens were machined into standard Charpy impact samples with dimensions of 10 mm × 10 mm × 55 mm, as shown in Figure 3. The impact performance was evaluated using a JBN-300B pendulum impact testing machine (Jinan Lichi Testing Equipment Co., Ltd., Jinan, China). For each condition, five parallel samples were tested to ensure the reproducibility and accuracy of the experimental data.

After heat treatment, metallographic specimens were mechanically ground, polished and etched with a 4 vol.% nital (nitric acid in ethanol) for 15 s to reveal the microstructure. Microstructural observations were performed using a scanning electron microscope (SEM, Thermo Scientific Apreo 2C) (Thermo Fisher Scientific Inc., Waltham, MA, USA) operated at an accelerating voltage of 25 kV. For orientation and grain boundary analysis, specimens were electropolished in a 5% perchloric acid–ethanol solution at 15 V for 50 s. The crystal orientation and grain boundary distribution of the experimental steel were analyzed by means of an electron backscatter diffraction (EBSD) detector (EDAX Velocity Super) (EDAX LLC, Mahwah, NJ, USA), and the data acquisition step was 0.2 μm. Standard cleaning steps such as grain confidence index (CI) standardization and grain dilation are adopted to remove isolated noise. At last, the TSL OIM software (OIM Analysis 9.1) is used to reconstruct the grain, and the threshold angle of grain boundary is 15° (that is, the boundary with orientation difference > 15° is regarded as the grain boundary for dividing the grain). Then the equivalent circle diameter of each reconstructed grain is calculated and the average value is counted. Precipitation behavior was characterized via high-resolution field-emission transmission electron microscopy (TEM, Talos F200X) (Thermo Fisher Scientific Inc., Hillsboro, OR, USA). TEM foils were prepared by cutting Φ5 mm discs from the specimens, mechanically thinning them to 0.5 mm, and subsequently processing them via the electrolytic twin-jet method combined with ion thinning. The electrolyte comprised 5% perchloric acid and 95% ethanol, operated at 20 V and maintained at −30 °C. The morphology, crystal structure, and chemical composition of the precipitates were further analyzed using a Bruker energy-dispersive X-ray spectrometer (EDS). Quantitative analysis of the structure, quantity, and chemical composition of precipitates was performed through physical and chemical phase analysis. The precipitate was extracted by electrolysis using 10 g/L tetramethylammonium chloride + 10% ethyl acetone methanol (current 0.03–0.05 A/cm^2^, temperature −5–0 °C). After collection, cleaning, and drying, the structure of the precipitate was determined using a Malvern Panalytical X’Pert CPD X-ray spectrometer (Malvern Panalytical B.V., Almelo, The Netherlands) (Cu target Kα at 40 V and 40 mA). The elemental content in the precipitate was measured using inductively coupled plasma atomic emission spectrometry (ICP-AES). M_3_C particles were dissolved in a 5% to 10% (*v*/*v*) hydrochloric acid ethanol solution while retaining MC particles. Then, according to GB/T 13221 standards [34], a powder X-ray spectrometer (PANalytic X’Pert CPD)/Kratky small angle scattering angle measuring instrument (Malvern Panalytical B.V., Almelo, The Netherlands) was used to determine the size distribution of the MC phase using a Cu target Kα at 40 V and 40 mA. For the calculation of dislocation density, a Bruker D8 Advance X-ray diffractometer (XRD) was used, with Co target Kα radiation (λ = 0.17902 nm), tube voltage of 40 kV, tube current of 40 mA, scanning range of 2*θ* = 30–90°, scanning step size of 0.02°, and scanning speed of 0.5°/min. The diffraction peaks obtained from standard annealed silicon powder under the same testing conditions were used as the instrument broadening curve. For each sample peak, the background was first deducted, followed by peak shape fitting using the Pearson VII function to obtain the measured integral width *β*_obs_. The instrument broadening *β*_inst_ is obtained from the integral width of the corresponding peak of silicon powder. The physical broadening *β* is approximated by the formula:

(1) $$\beta =(\beta_{\mathrm{obs}}^{2}-\beta_{\mathrm{inst}}^{2}{)}^{1/2}$$

The Williamson–Hall method is used for broadening separation: *β*cosθ = Kλ/D + 4εsin*θ*. Among them, *β* is the physical broadening (radians), *θ* is the Bragg angle, K = 0.9, λ = 0.17902 nm. Linear fitting is performed on the obtained experimental results, and the crystal block size d is obtained from the intercept of the fitted line. The dislocation density ρ can be calculated according to the formula ρ = 14.4ε^2^/b^2^, where b is the Burgers vector (for BCC iron, b = a√3/2, a is the lattice constant 0.2866 nm, so b ≈ 0.248 nm). The coefficient of 14.4 is derived from the theoretical relationship between dislocation strain field and X-ray line broadening, and is applicable to randomly distributed dislocations [35].

## 3. Results

### 3.1. Mechanical Properties

**Figure 4:**
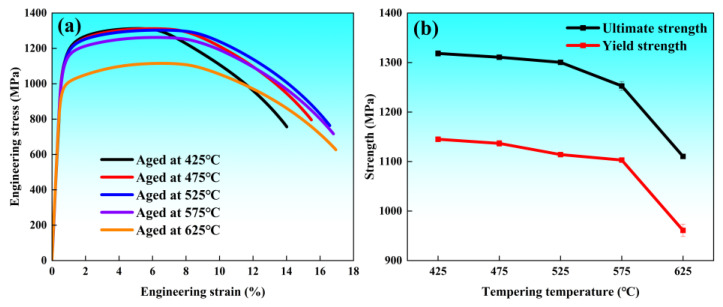

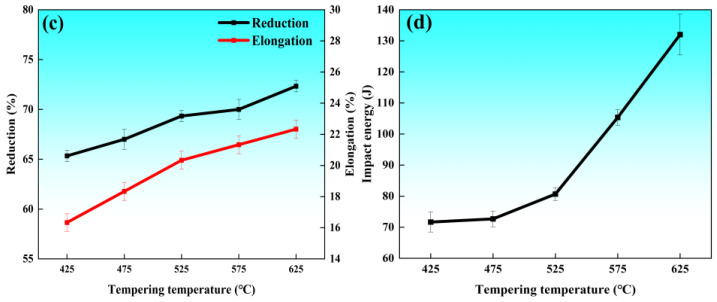
Mechanical properties of Cr-Ni-Mo-V steel at different tempering temperatures: (**a**) engineering stress–strain curves; (**b**) tensile properties; (**c**) elongation and reduction in area; (**d**) impact energy.

Figure 4 illustrates the room-temperature mechanical properties of the Cr-Ni-Mo-V steel following quenching and tempering at various temperatures, establishing the correlation between strength, plasticity, and toughness as a function of tempering temperature. Figure 4a presents the engineering stress–strain curves. The peak stress remains sustained at a high level for specimens tempered between 425 °C and 525 °C, whereas the ultimate tensile strength declines markedly at tempering temperatures of 575 °C and 625 °C. Notably, a pronounced yield plateau was observed during low-to-medium temperature tempering (425–475 °C), which gradually diminished and transitioned to continuous yielding behavior at higher temperatures (575–625 °C). Figure 4b depicts the evolution of strength. The results demonstrate that both tensile strength and yield strength decrease as the tempering temperature rises. The tensile strength exhibits a plateau of approximately 1300 MPa between 425 °C and 525 °C before descending rapidly. Similarly, the yield strength remains stable at around 1100 MPa from 425 °C to 575 °C, after which a substantial drop occurs. The plasticity parameters, namely, the reduction in area (Z) and elongation (A), are shown in Figure 4c. Both indicators show a positive correlation with the tempering temperature: elongation increases from 16% at 425 °C to 22% at 625 °C, while the reduction in area rises from 65% to 72% across the same range. Figure 4d highlights the variation in Charpy V-notch impact energy. The impact energy increased progressively with temperature; the growth was relatively gradual between 425 °C and 525 °C (maintaining 70–80 J), but accelerated significantly at higher temperatures. Compared to the 525 °C condition, the impact energy surges by 25 J to reach 105 J at 575 °C, and further culminates at 132 J at 625 °C. Notably, the yield strength/tensile strength ratio of the specimen tempered at 575 °C shows an obvious change compared with other tempering temperatures, which is consistent with the secondary hardening effect induced by the massive precipitation of MC-type alloy carbides at this temperature. This phenomenon further confirms that the precipitation behavior of MC carbides becomes the core factor regulating the strength evolution of the experimental steel in the medium-to-high tempering stage.

### 3.2. Microstructure Evolution During Tempering

**Figure 5:**
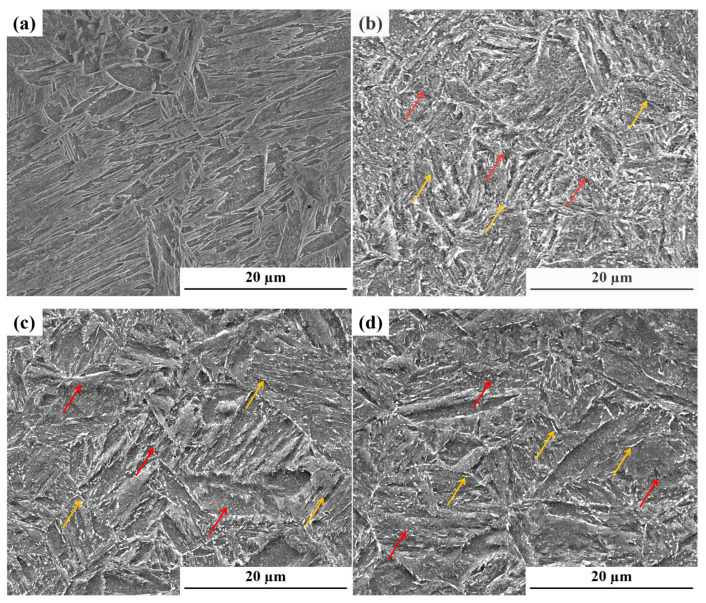
SEM microstructures of Cr-Ni-Mo-V steel at different tempering temperatures: (**a**) as-quenched; (**b**) 425 °C; (**c**) 525 °C; (**d**) 625 °C. The red arrow represents the spherical precipitate phase, and the yellow arrow represents the sheet-like precipitate phase.

Figure 5 illustrates the SEM micrographs of Cr-Ni-Mo-V steel in the as-quenched state and after tempering at three representative temperatures (425 °C, 525 °C, and 625 °C). Figure 5a displays the as-quenched microstructure, characterized by a typical hierarchical lath martensitic morphology. The prior austenite grain boundaries (PAGBs) are well-defined, and the martensitic laths are densely and uniformly arranged with high aspect ratios and sharp, distinct boundaries. The matrix material maintains significant lattice distortion and high dislocation density, accompanied by the presence of locally clustered coarse undissolved carbides, which is consistent with the microsegregation-induced carbide precipitation behavior described in Section 2.1. Upon tempering at 425 °C (Figure 5b), the martensitic lath boundaries begin to blur, accompanied by the appearance of fine, dispersed carbide particles at the lath and grain boundaries. The absence of significant coarsening or aggregation suggests that only localized recovery and initial carbide nucleation occur during low-temperature tempering. As the temperature rises to 525 °C (Figure 5c), the lath features become increasingly indistinct, with localized coalescence of lath packets and a moderate increase in lath width. The microstructure evolves into tempered martensite, characterized by a significant increase in carbide precipitation and a tendency for accumulation at grain and lath boundaries. This indicates a transition from the initial non-equilibrium state toward a more stable metastable condition. When the tempering temperature is further raised to 625 °C (Figure 5d), the martensite lath structure basically disappears. At this time, the carbide coarsens significantly, and its shape gradually changes from needle-like to nearly spherical, and it is evenly distributed at the matrix and subgrain boundaries. On the whole, with the increase in tempering temperature, martensite lath gradually degenerates, and precipitated phases gradually increase and grow.

**Figure 6:**
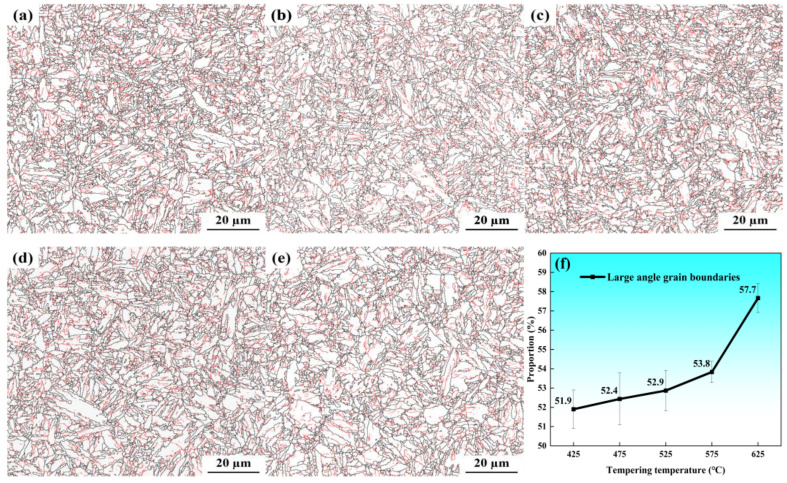
EBSD microstructures of Cr-Ni-Mo-V steel at different tempering temperatures: (**a**) 425 °C; (**b**) 475 °C; (**c**) 525 °C; (**d**) 575 °C; (**e**) 625 °C; (**f**) proportion of HAGBs.

Figure 6 quantitatively characterizes the evolution of grain boundary distributions and average effective grain size of the Cr-Ni-Mo-V steel tempered from 425 °C to 625 °C, as analyzed via electron backscatter diffraction (EBSD). In the orientation maps, high-angle grain boundaries (HAGBs, misorientation > 15°) are delineated by black lines, while low-angle grain boundaries (LAGBs, misorientation 2–15°) are highlighted in red. As shown in Figure 6a–c, the microstructures tempered at 425 °C to 525 °C exhibit a remarkably high density of red LAGBs, reflecting the significant retention of martensitic substructures and high dislocation densities inherited from the as-quenched state. At these temperatures, the microstructure consists of fine, interwoven martensitic laths, where the boundaries of packets and blocks are clearly visible, and the interior regions of laths are densely populated with subgrain boundaries (i.e., LAGBs). With the further increase in tempering temperature (Figure 6d,e), the density of red LAGBs decreases significantly. This is attributed to two main processes during tempering: on the one hand, enhanced dislocation annihilation and rearrangement via recovery reduce the dislocation content that constitutes LAGBs; on the other hand, the replacement of cementite by more thermally stable alloy carbides weakens the Zener pinning effect on lath and subgrain boundaries, enabling the migration and coalescence of subgrains. This trend is quantitatively substantiated in Figure 6f: the relative proportion of HAGBs in total grain boundaries increases monotonically with tempering temperature, rising from 51.9% at 425 °C to 53.8% at 575 °C, followed by a sharp increase to 57.7% at 625 °C. It should be emphasized that this increase in HAGB proportion is not caused by the growth of absolute HAGB content, but by the significant reduction in total grain boundary length per unit area due to the massive disappearance of LAGBs. Furthermore, statistical analysis indicates that the average effective grain size of the tested steel remains remarkably stable across the entire investigated tempering range, measured at 1.97 ± 0.02 μm. Considering the limited statistical sample size in the effective grain size statistical analysis, along with the local inhomogeneity induced by undissolved carbides, the measured average effective grain size will exhibit non-negligible statistical errors. These statistical errors have been fully accounted for in subsequent Hall–Petch strengthening calculations and yield strength model predictions. It should be pointed out here that the effective grain size is defined as the equivalent circle diameter of the region surrounded by HAGBs (i.e., the average size of martensite blocks), as HAGBs act as effective barriers to dislocation slip and plastic deformation, while LAGBs only serve as substructures within the same effective grain with limited hindrance to dislocation motion. The stable effective grain size further confirms that the absolute content of HAGBs does not change significantly during tempering, and only the relative proportion of HAGBs in total grain boundaries increases with the elimination of LAGBs.

**Figure 7:**
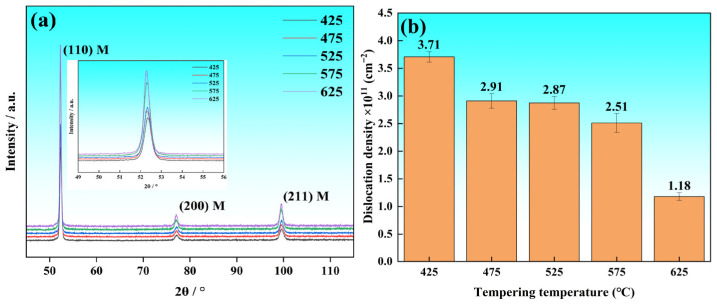
XRD characterization of Cr-Ni-Mo-V steel at different tempering temperatures: (**a**) XRD patterns; (**b**) evolution of dislocation density.

Figure 7 displays the refined XRD characterization and the corresponding evolution of dislocation density for the Cr-Ni-Mo-V steel across the tempering range of 425 °C to 625 °C. As shown in the magnified (110) crystal plane diffraction peaks in revised Figure 7a, all specimens tempered at different temperatures exhibit a single sharp diffraction peak corresponding to the body-centered cubic (BCC) α-Fe matrix, with no discernible peak splitting, shoulder peaks or abnormal peak shifts related to the body-centered tetragonal (BCT) structure. This confirms that the matrix of the tested steel maintains a single BCC structure in the entire tempering temperature range, and no long-range ordered BCT structure is formed. The lattice constant of the matrix is calculated based on the precise peak position of the (110) diffraction peak, which decreases from 0.2871 nm at 425 °C to 0.2866 nm at 625 °C, gradually approaching the standard lattice constant of pure α-Fe (0.2866 nm). This trend indicates that the content of supersaturated solid solution carbon in the matrix decreases continuously with the increase in tempering temperature, which is consistent with the precipitation behavior of carbides revealed by physicochemical phase analysis. No characteristic peaks of retained austenite or other secondary phases are detected within the resolution limits of XRD for all samples. The quantitative analysis of peak broadening, based on the Williamson–Hall method, is presented in Figure 7b, illustrating the two-stage evolution characteristic of dislocation density with tempering temperature. The results demonstrate that the dislocation density maintains a high-stability platform at 425–525 °C, with only a slight decrease from 3.71 × 10^11^ cm^−2^ at 425 °C to 2.87 × 10^11^ cm^−2^ at 525 °C. When the tempering temperature rises to 575 °C and 625 °C, the dislocation density decreases significantly, dropping sharply to 1.18 × 10^11^ cm^−2^ at 625 °C. The high dislocation density at 425 °C is mainly inherited from the intense lattice distortion and plastic deformation during the martensitic transformation, and its retention is closely related to the pinning effect of carbon segregation and cementite precipitation on dislocations. It should be pointed out here that the presence of a small amount of undissolved carbides in the steel will lead to local lattice distortion, which has a certain impact on the peak broadening and the calculated dislocation density, and this factor has been considered in the subsequent discussion of strengthening mechanisms.

### 3.3. Precipitation Analysis

**Figure 8:**
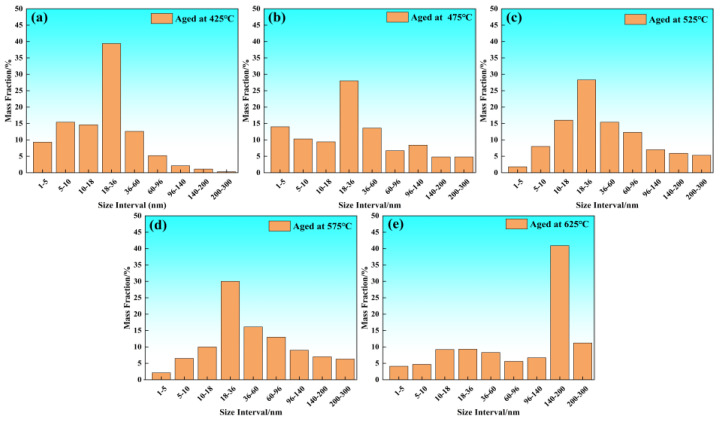
Size distribution of MC-type carbides in Cr-Ni-Mo-V steel at different tempering temperatures: (**a**) 425 °C; (**b**) 475 °C; (**c**) 525 °C; (**d**) 575 °C; (**e**) 625 °C.

Figure 8 quantitatively illustrates the size distribution evolution of MC-type carbides in the tested Cr-Ni-Mo-V steel within the tempering temperature range of 425 °C to 625 °C, while Table 2 and Table 3 summarize the mass fractions of constituent elements within the MC phase and M_3_C phase at each tempering temperature, respectively. The precipitation behavior of MC and M_3_C carbides exhibits a distinct three-stage evolution characteristic with increasing tempering temperature, as detailed below. In the low-to-medium tempering temperature range of 425 °C to 525 °C, M_3_C-type cementite is the dominant newly precipitated secondary phase, while no massive precipitation of new MC-type carbides occurs in the matrix. As shown in Table 3, the total mass fraction of M_3_C phase increases significantly from 0.493 wt.% at 425 °C to 1.837 wt.% at 525 °C, which is the most prominent microstructural evolution in this tempering stage. For MC-type carbides in this temperature range, the size distribution shown in Figure 8a–c exhibits no statistically significant difference between 425 °C, 475 °C and 525 °C. The MC particles are dominated by pre-existing undissolved carbides inherited from the as-quenched state, with only a negligible amount of new MC precipitates formed in the matrix. At 425 °C tempering, the MC phase is almost pure VC with a total mass fraction of only 0.021 wt.%, and the particles are mainly distributed in the size range of 18–36 nm (mass fraction close to 40%), while coarse particles larger than 96 nm are extremely rare. When the tempering temperature is raised to 475 °C and 525 °C, the overall size distribution of the MC phase remains nearly unchanged, with the 18–36 nm particles still occupying the dominant proportion, and only slight fluctuation occurs in the proportion of ultrafine particles in the 1–18 nm range. Meanwhile, the content of Cr, Mo, and V in the MC phase shows a slight increase, reflecting only limited compositional adjustment of the pre-existing MC particles, rather than massive nucleation and growth of new MC precipitates. Correspondingly, the total mass fraction of alloying elements in the MC phase increases slightly from 0.021 wt.% at 425 °C to 0.190 wt.% at 525 °C, which is within the statistical fluctuation range of the physicochemical phase analysis method, and cannot confirm the massive precipitation of new MC carbides in this temperature interval. The local inhomogeneity of these undissolved carbides is the main cause of statistical fluctuations in the measured size distribution of MC particles. When the tempering temperature further increases to 575 °C, a significant transition in precipitation behavior occurs. The size distribution of MC carbides shifts markedly towards larger sizes, with the proportion of medium-sized particles between 36–96 nm increasing significantly and particles larger than 96 nm beginning to appear. The total mass fraction of the MC phase surges to 0.377 wt.%, with the mass fractions of V and Mo reaching 0.135 wt.% and 0.123 wt.%, respectively, indicating the substantial precipitation of new MC carbides from the supersaturated matrix and the onset of significant coarsening via the Ostwald ripening mechanism. This precipitation behavior of MC carbides is consistent with the secondary hardening effect reflected by the yield strength/tensile strength ratio change in Figure 4b at 575 °C. Concurrently, the total mass fraction of M_3_C phase decreases slightly to 1.436 wt.%, indicating that the M_3_C phase begins to dissolve and provide carbon and alloying elements for the precipitation and growth of MC carbides. When the tempering temperature reaches 625 °C, the size distribution of MC carbides changes drastically, with coarse particles in the range of 140–200 nm dominating (over 40% mass fraction). The total mass fraction of the MC phase reaches 0.414 wt.%, and the contents of V and Mo further increase to their peak values (0.146 wt.% and 0.135 wt.%, respectively), meaning that the MC carbides have entered the stage of sufficient coarsening. At this temperature, the M_3_C phase has been almost completely dissolved or replaced by alloy carbides, and MC-type carbides become the only dominant secondary precipitate phase in the matrix.

**Figure 9:**
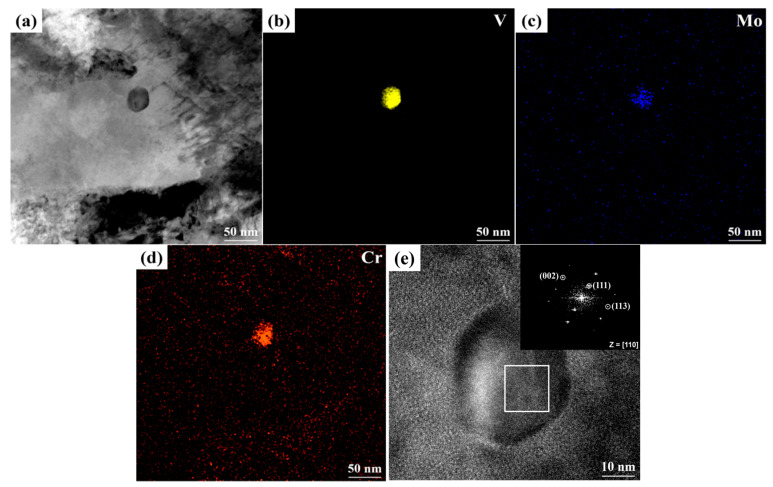
TEM characterization of precipitates in Cr-Ni-Mo-V steel after tempering at 525 °C: (**a**) bright-field (BF) image; (**b**–**d**) elemental mapping of V, Mo, and Cr; (**e**) HRTEM image and the corresponding FFT pattern of the precipitate.

Figure 9 provides a comprehensive characterization of the morphology, chemical composition, and crystal structure of the carbides in Cr-Ni-Mo-V steel after tempering at 525 °C, utilizing transmission electron microscopy and energy-dispersive spectroscopy. As shown in Figure 9a, numerous nanoscale precipitates with near-spherical or elliptical morphologies are dispersed within the matrix, possessing an average diameter of approximately 43 nm. To identify the chemical nature of these particles, elemental mapping was conducted (Figure 9b–d), revealing a pronounced enrichment of V, Mo, and Cr. The concentration of V is particularly significant (Figure 9b), confirming that the precipitates are complex (V, Mo, Cr)C carbides. This elemental distribution aligns perfectly with the results obtained from the physicochemical phase analysis. A high-resolution TEM (HRTEM) image is presented in Figure 9e, clearly resolving the lattice fringes within a representative precipitate. Fast Fourier Transform (FFT) analysis of the indexed region in the HRTEM image produces a diffraction pattern characteristic of a face-centered cubic (FCC) structure. By means of diffraction pattern analysis, combined with the evidence of morphology, composition and crystallography, these precipitates are identified as MC carbide.

## 4. Discussion

### 4.1. The Effect of Tempering Temperature on the Microstructure and Precipitated Phases of Cr-Ni-Mo-V Steel

With a fixed tempering holding time of 2 h, this study observes that the dislocation density decreases progressively as the tempering temperature rises. The fundamental driver for this phenomenon is the recovery process, during which dislocations undergo rearrangement and annihilation to alleviate internal stresses [36]. At relatively low tempering temperatures, although thermal activation initiates short-range carbon diffusion and segregation, dislocation mobility remains severely constrained. This restriction stems from the potent interaction between interstitial carbon atoms and dislocation cores, coupled with the solute drag effect exerted by substitutional atoms, particularly Mo and V. Consequently, at this stage, long-range climb and cross-slip are energetically unfavorable, leading to a restricted reduction in dislocation density [37]. However, as the tempering temperature further increases, the system gains sufficient thermal activation energy to overcome the pinning effects of solute atoms and interstitial clusters. Elevated temperatures markedly enhance the diffusion kinetics of vacancies, facilitating the cross-slip and climb of dislocations. Concurrently, the increased vacancy concentration in the iron matrix provides the necessary flux for dislocations of opposite signs to attract and mutually annihilate. Meanwhile, dislocations of the same sign tend to rearrange into more energetically stable configurations, such as subgrain boundaries. This collective process significantly dissipates the lattice strain energy, ultimately resulting in a pronounced decline in dislocation density within the matrix.

On the premise of a fixed holding time of 2 h, this study observes a distinct two-stage evolution characteristic of dislocation density: a stable high-value platform at 425–525 °C, followed by a sharp decline at 575–625 °C. This evolution law is essentially controlled by the coupling effect of carbon precipitation behavior, solute segregation at dislocations, and the Zener pinning effect of secondary phases during tempering. At the tempering temperature range of 425–525 °C, M_3_C-type cementite is the dominant newly precipitated secondary phase, which preferentially nucleates and grows at lath boundaries and dislocation substructures. On the one hand, the precipitation of cementite consumes most of the supersaturated carbon in the matrix, leading to an extremely low concentration of carbon in solid solution. This prevents the formation of a long-range ordered BCT structure, which is consistent with the XRD results where no characteristic peaks of the BCT structure are detected. On the other hand, the fine and dispersed cementite particles and the undissolved MC carbides exert a strong Zener pinning effect on dislocations and lath boundaries, which severely hinders the climb, cross-slip and annihilation of dislocations [33]. Meanwhile, a small amount of carbon that has not been precipitated forms Cottrell atmospheres through short-range diffusion and segregates around dislocation cores, which further enhances the pinning effect on dislocations, inhibits the recovery and annihilation of dislocations, and reduces the mobility of dislocations. This is the core reason why the tested steel still maintains an extremely high dislocation density after tempering at 425 °C, and the dislocation density does not decrease significantly in the range of 425–525 °C. At this stage, only limited short-range rearrangement of dislocations occurs, and no large-scale annihilation process is triggered, so the dislocation density remains at a high level. When the tempering temperature rises to 575–625 °C, the diffusion kinetics of substitutional alloying elements are significantly enhanced, and the M_3_C cementite formed at low temperature gradually dissolves and is replaced by more thermally stable MC-type alloy carbides. The dissolution of fine cementite particles eliminates the strong pinning effect on dislocations, while the coarsening of MC carbides significantly increases the inter-particle spacing, leading to a drastic weakening of the overall Zener pinning effect on dislocations and lath boundaries. Under this condition, dislocations gain sufficient thermal activation energy and mobility to undergo large-scale rearrangement, climb and annihilation [34]. Meanwhile, the weakening of grain boundary pinning effect promotes the coalescence and coarsening of martensite laths, and the sweeping of lath boundaries during the coarsening process further promotes the annihilation of dislocations. This collective process ultimately leads to the sharp decrease in dislocation density at 575–625 °C.

The tempering process concurrently facilitates the elimination of low-angle grain boundaries within the martensitic matrix [38]. Fundamentally, the thermal activation energy provided by tempering drives the microstructure toward a state of thermodynamic equilibrium. During the low-to-medium temperature tempering stages, the available activation energy is relatively limited. At this point, the fine, dispersedly distributed carbides exert a potent Zener pinning effect on dislocation glide. Consequently, LAGBs—acting as primary regions for dislocation accumulation—are stabilized by this pinning action. During this stage, the microstructural evolution is dominated by localized recovery, wherein subgrain boundaries are only marginally refined through dislocation rearrangement. There is no substantial increase in misorientation or significant transformation of grain boundary types; thus, the proportion of LAGBs declines only gradually. However, as the tempering temperature further increases, the elevated thermal activation energy not only accelerates the diffusion kinetics of carbon atoms but also promotes the coarsening and aggregation of carbide particles. This increase in inter-particle spacing significantly attenuates the pinning force acting on dislocations and subgrain boundaries. Facilitated by the continuous climb and cross-slip of dislocations, subgrain boundaries begin to migrate and merge through subgrain coalescence, leading to a progressive increase in subgrain size. Concurrently, the misorientation across these boundaries intensifies; once the misorientation angle exceeds the 15° threshold, these LAGBs transition into high-angle grain boundaries (HAGBs). This mechanistic shift results in the pronounced reduction in the fraction of low-angle grain boundaries observed at higher temperatures.

The gradual evolution of secondary carbide phases with increasing tempering temperature represents the most critical microstructural feature in the tested Cr-Ni-Mo-V steel, which is dominated by the precipitation, dissolution and coarsening behavior of M_3_C-type cementite and MC-type alloy carbides in different tempering stages. At the low-to-medium tempering temperature range of 425 °C to 525 °C, the reduction in thermodynamic stability of the supersaturated solid solution mainly drives the short-range diffusion of carbon atoms, leading to the massive nucleation and growth of M_3_C-type cementite at lath boundaries and dislocation substructures. In this temperature range, the long-range diffusion of substitutional alloying elements (V, Mo, Cr) is severely limited by the low thermal activation energy, so only a negligible amount of MC-type carbides precipitate from the matrix, and the pre-existing undissolved MC particles inherited from the as-quenched state only undergo limited compositional adjustment without significant coarsening. Therefore, the evolution of M_3_C cementite is the dominant precipitation behavior in the low-to-medium tempering stage, which exerts a non-negligible influence on the mechanical properties of the steel in this temperature range. With the tempering temperature rising to 575 °C and 625 °C, sufficient thermal activation energy significantly enhances the diffusion kinetics of substitutional alloying elements. At this stage, the M_3_C cementite formed at low temperature gradually dissolves, releasing carbon and alloying elements into the matrix, which promotes the massive nucleation and growth of MC-type carbides from the supersaturated matrix. According to TEM and physicochemical phase analyses, vanadium (V) serves as the primary alloying element in the MC phase, followed by Mo and Cr. As the tempering temperature increases, the system moves toward minimizing its total interfacial energy. Smaller MC particles, characterized by higher chemical potential due to their greater curvature, become thermodynamically unstable relative to larger ones. This establishes a chemical potential gradient that drives the solute atoms to diffuse from the smaller particles toward the surfaces of the larger ones. Consequently, these MC precipitates undergo progressive coarsening via the Ostwald ripening mechanism, wherein smaller particles gradually dissolve and their constituent solute atoms diffuse through the matrix to deposit onto larger particles, leading to a significant increase in the average particle size. At 625 °C, the M_3_C phase is almost completely dissolved, and MC-type carbides become the only stable secondary precipitate phase in the matrix [39].

### 4.2. The Effect of Tempering Temperature on the Mechanical Properties of Cr-Ni-Mo-V Steel

The microstructural evolution of Cr-Ni-Mo-V steel during tempering exerts a profound influence on its mechanical properties [40]. On the premise of keeping the temperature for 2 h, as the tempering temperature rises, enhanced dislocation mobility and rearrangement facilitate the recovery of high-density dislocations, which subsequently aggregate into lower-energy configuration [41]. This reduction in dislocation density diminishes the extent of dislocation entanglement and the overall resistance to motion within the matrix, thereby lowering the energy barrier for plastic deformation and leading to a decline in strength [42]. Furthermore, the increase in tempering temperature triggers a transition in dislocation configurations from complex, disordered entanglements to ordered dislocation lines and wall structures. This transformation reduces the impedance caused by dislocation intersections and significantly extends the dislocation mean free path. Consequently, dislocations can undergo large-scale glide across martensitic laths, shifting the deformation mode from localized strain concentration to a more globally uniform deformation, which markedly enhances ductility [43]. Simultaneously, the coarsening of martensitic laths at elevated temperatures leads to a reduction in the total surface area of lath boundaries. This decrease in the density of interfaces per unit volume reduces the contribution of boundary strengthening. However, these coarsened laths host more readily activated slip systems, facilitating uniform intra-lath deformation through dislocation glide. This mitigates deformation incompatibility between adjacent laths, further improving plasticity [44]. For Cr-Ni-Mo-V steel, precipitation strengthening is a decisive factor governing the trade-off between strength and plasticity. Throughout the tempering process, the MC phase evolves via the Ostwald ripening mechanism. While fine carbides provide potent pinning of dislocation motion—contributing to high strength—they also induce significant stress concentration at the carbide/matrix interfaces, which can be detrimental to plasticity. As the tempering temperature increases, the carbides coarsen, and the associated lattice mismatch and interfacial strain energy significantly decrease. This relief of interface stress concentration promotes a transition toward improved plasticity and toughness [45].

It should be noted that the evolution of M_3_C-type cementite plays a dominant role in the tensile properties of the steel tempered at 425 °C, 475 °C and 525 °C. The massive precipitation of cementite in this temperature range not only influences the dislocation motion and work-hardening behavior of the matrix, but also leads to the stable maintenance of yield strength and tensile strength, which corresponds to the slight change in yield strength/tensile strength ratio in Figure 4b for the three lowest tempering temperatures. When the tempering temperature rises to 575 °C, the significant precipitation of MC-type alloy carbides induces a secondary hardening effect, which is consistent with the obvious change in yield strength/tensile strength ratio at this temperature, and becomes the core factor regulating the strength evolution in the medium-to-high tempering stage. To more clearly quantify the contribution of different strengthening mechanisms to the strength, the yield strength of Cr-Ni-Mo-V steel is determined by considering four main strengthening components: grain boundary strengthening, precipitation strengthening, dislocation strengthening, and solid solution strengthening. The calculation is performed using root sum square superposition [46]:

(2) $$\sigma_{y}=\sigma_{0}+\sigma_{\mathrm{ss}}+\sqrt{\sigma_{d}^{s}+\sigma_{g}^{2}+\sigma_{p}^{2}}$$

where *σ*_0_ is the lattice resistance, taken as 40 MPa here. σ_ss_ is the solid solution strengthening component; σ_d_ is the dislocation strengthening component; *σ_g_* is the grain boundary strengthening component; and *σ_p_* is the precipitation strengthening component.

The solid solution strengthening component can be calculated by the following Equation (3) [47]:

(3) $$\sigma_{\mathrm{ss}}=k_{C}{\left[C\right]}^{\frac{1}{2}}+k_{M}\left[M\right]$$

where *k_C_* is a constant, taken as 1722.5 [48]; [M] is the mass percentage of C dissolved in the matrix; kM is the mass percentage of weak solid solution strengthening elements; where Mn, Mo, V, and Cr are taken as 11, 32, 3, and −30 [49], respectively; and [M] is the mass fraction of the solid solution elements in the matrix.

The dislocation strengthening component can be calculated by the following Equation (4) [50]:

(4) $$\sigma_{d}=\alpha Gb\sqrt{\rho }$$

where *α* is a constant related to the crystal structure, taken as 0.3; G is the shear modulus, taken as 81 GPa; *b* is the Burgers vector, taken as 0.248 nm; *ρ* is the dislocation density, with units of m^−2^.

The grain boundary strengthening component can be calculated using the Hall–Petch Equation (5) [51]:

(5) $$\sigma_{g}=kd^{-\frac{1}{2}}$$

where *k* is the proportionality constant, taken as 14 MPa/mm^0.5^, and *d* is the effective grain size, which refers to the minimum grain size formed by the grain boundaries that hinder dislocation slip motion and cause dislocation pile-up. Here *d* is 1.97 × 10^−3^ mm. It should be pointed out here that in this study, because the effective grain size is only determined by the network of HAGBs, the disappearance or transformation of LAGBs during tempering mainly affects the dislocation structure in subgrain, and does not significantly change the grain skeleton outlined by HAGBs. HAGBs have been formed in the as-quenched state, and the grain size has not grown obviously in the subsequent tempering, so the grain size defined by HAGBs is basically unchanged. At the same time, although the increase in the proportion of HAGBs mainly comes from the transformation of some LAGBs into HAGBs, which increases the total length/area of HAGBs, it does not increase the area surrounded by a single HAGB (that is, the effective grain size). Therefore, in the calculation, the effective grain size at each tempering temperature is considered to be consistent.

The precipitation strengthening increment can be calculated using the following Equation (6) [52]:

(6) $$\sigma_{p}=8.995\times 10^{3}\frac{f^{\frac{1}{2}}}{d}\mathrm{In}2.417d$$

where *d* is the size of the precipitate phase, in nm; *f* is the volume fraction of the precipitate phase, and *f* is calculated using Equation (7):

(7) $$f=f_{m}\frac{\rho_{Fe}}{100\rho_{MC}}$$

where *ρ_Fe_* is the density of iron, taken as 7.875 g/cm^3^; *ρ_MC_* is the density of the MC phase, and *f_m_* is the mass fraction of the MC phase. The MC phase in Cr-Ni-Mo-V steel is calculated using the weighted average density of the precipitates, with the specific results shown in Table 4.

**Figure 10:**
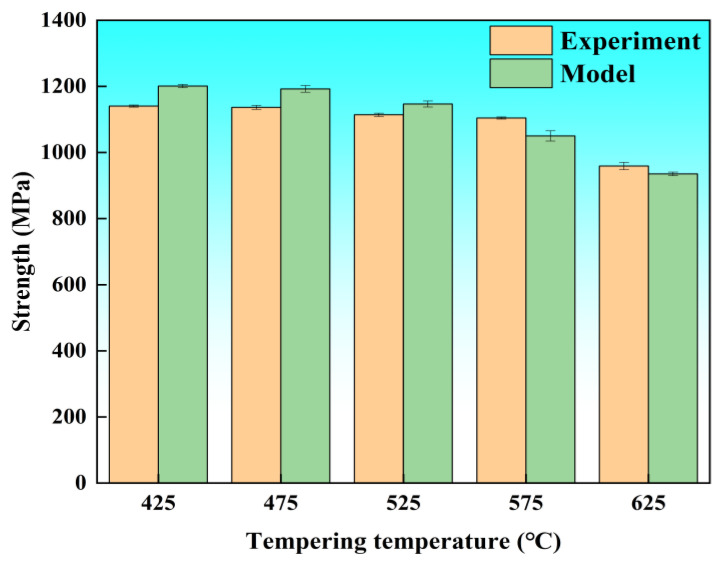
Comparison between calculated and measured yield strength values of Cr-Ni-Mo-V steel.

The calculated and measured values of yield strength of the tested steel at different tempering temperatures are summarized in Table 5 and Figure 10. The calculation results show good consistency with the experimental data at 575 °C and 625 °C, while a non-negligible deviation exists between the model-predicted values and the experimental results at 425 °C, 475 °C and 525 °C. Specifically, the model overestimates the magnitude of yield strength reduction with increasing tempering temperature in the low-to-medium tempering range. This deviation is mainly attributed to the fact that the current strengthening model does not take into account the precipitation strengthening contribution from M_3_C-type cementite, which is the dominant secondary phase in the 425–525 °C tempering interval. The neglect of cementite strengthening leads to an overestimation of the contributions from MC precipitation strengthening and solid solution strengthening in the low-to-medium tempering range, as shown in Table 5. In addition to the above deviation, other core sources of error include the following aspects: (1) the statistical errors of grain size and MC carbide characteristics caused by the limited statistical sample size, which directly affect the calculation accuracy of grain boundary strengthening and precipitation strengthening, and the error range has been added to the relevant result figures and calculation results; (2) the fact that the calculation of solid solution strengthening is based on the average content of solute elements in the matrix, without considering the non-uniform distribution of solid solution carbon atoms (i.e., the formation of Cottrell atmospheres around dislocations), which will also lead to a slight deviation between the calculated value and the experimental value; (3) the assumption deviation of the precipitation strengthening model, and the absence of coupling effects between different strengthening mechanisms; (4) the error transfer from the measured dislocation density and lattice constant to the final yield strength prediction, which has been considered in the error bars of the predicted yield strength in Figure 10. All the above error factors have been systematically considered in the quantitative calculation of strengthening mechanisms, and the error range has been included in the deviation analysis between the model predicted values and the experimental results.

The microstructural evolution during tempering exerts a fundamental influence on the impact toughness of the tested steel. For Charpy impact loading in this study, the total absorbed impact energy is dominated by the energy consumed prior to the formation of a critically sized, self-propagating crack (i.e., crack initiation energy), rather than the energy consumed during crack propagation. Accordingly, the improvement in impact toughness with increasing tempering temperature is mainly attributed to the significant elevation of the crack initiation threshold, which is controlled by the evolution of dislocation structures, martensitic lath boundaries, and precipitated carbides, as detailed below. The high-density dislocation structure inherited from the as-quenched state is a major source of crack initiation under impact loading. High-density dislocations are prone to form pile-up groups at grain boundaries and lath boundaries under high-rate impact deformation, causing severe local stress concentration, which lowers the crack initiation threshold and provides preferential sites for crack nucleation. During tempering, dislocation recovery via slip, climb, and annihilation of dislocations with opposite signs greatly reduces the matrix dislocation density, eliminating the stress concentration induced by dislocation pile-up. With increasing tempering temperature, the dislocation density decreases continuously, and the dislocation cell structures inherited from quenching are gradually eliminated, resulting in a more homogeneous matrix with far fewer stress concentration sites, thus significantly reducing the probability of crack initiation [53]. In the quenched and low-temperature tempered steel, a large number of fine martensitic lath boundaries exist, and notable stress concentration occurs between adjacent laths due to deformation incompatibility under impact loading. Microcracks are easily initiated at these lath boundaries and can propagate rapidly along the interfaces, leading to low-energy fracture and low impact toughness. As the tempering temperature increases, the martensitic laths undergo recovery and coarsening, which reduces the number density of lath boundaries and alleviates the stress concentration between adjacent laths. This increases the difficulty of microcrack initiation at lath boundaries under impact loading, thus contributing to the improvement of impact toughness [54]. The precipitated carbides in the steel act as another primary type of crack initiation sites under impact loading. The elastic modulus mismatch between carbides and the iron matrix easily aggravates local stress concentration at the carbide/matrix interface, making these interfaces preferential sites for crack nucleation. Fine nanoscale carbides are prone to interfacial debonding under high-rate applied stress, which directly induces the formation of microcracks; meanwhile, the brittle carbides themselves may fracture under impact loading, further acting as initiation sites for crack propagation [55]. As the tempering temperature increases, the carbides undergo gradual coarsening and spheroidization, which significantly reduces the stress concentration at the carbide/matrix interface. This suppresses interfacial debonding and carbide fracture, reduces the number of effective crack initiation sites, and thus raises the crack initiation threshold of the steel [56]. It should be supplemented that the increase in the relative proportion of HAGBs during tempering is a result of the massive elimination of LAGBs, while the absolute content of HAGBs and the effective grain size defined by HAGBs remain stable throughout the tempering range. The reduction in LAGBs is driven by the recovery and rearrangement of dislocations, as well as the weakened Zener pinning effect when cementite is replaced by coarsened MC carbides, which reduces the total grain boundary length per unit area and thus increases the relative proportion of HAGBs. Although HAGBs can impose a certain hindrance to crack propagation, as reported in previous studies [57,58,59], this effect is not the dominant factor for the improvement of impact toughness in this work, given the unchanged effective grain size and the fact that the impact energy is mainly controlled by the crack initiation process rather than the crack propagation process.

## 5. Conclusions

In this work, the effects of different tempering temperatures on the microstructure evolution, carbide precipitation behavior and mechanical properties of Cr-Ni-Mo-V steel under the premise of holding for 2 h were systematically studied, and the following conclusions can be drawn:

1. As the tempering temperature increases from 425 °C to 625 °C, the tensile strength decreases from 1320 MPa to 1190 MPa, and the yield strength declines from 1140 MPa to 959 MPa, while the impact energy increases from 71 J to 132 J. Concurrently, the dislocation density drops from 3.71 × 10^11^ cm^−2^ to 1.18 × 10^11^ cm^−2^, and the fraction of high-angle grain boundaries rises from 51.9% to 57.7%.
2. The predominant precipitates in the Cr-Ni-Mo-V steel during tempering are MC-type carbides enriched with V, Mo, and Cr. At a tempering temperature of 425 °C, fine MC carbides smaller than 36 nm account for a dominant proportion of the population. As the tempering temperature rises, additional fine MC particles precipitate from the matrix; concurrently, the existing MC phase undergoes progressive coarsening. Upon reaching 625 °C, the size distribution shifts significantly, with large carbides exceeding 140 nm becoming the predominant fraction.
3. With the increase in tempering temperature, the decrease in dislocation density, the coarsening of carbides, and the elimination of martensitic lath boundaries in Cr-Ni-Mo-V steel are the main reasons for the decrease in strength and the improvement in toughness. Among them, the change in strength is mainly influenced by carbide precipitation strengthening, dislocation strengthening, fine grain strengthening, and solution strengthening in the matrix. The improvement in toughness is primarily attributed to the significant elevation of the crack initiation threshold, driven by the synergistic effect of the reduction in dislocation density, the alleviation of stress concentration at coarsened carbide interfaces, and the reduction in lath boundaries.
4. Considering both strength and toughness, 525 °C is the optimal tempering temperature for this Cr-Ni-Mo-V steel. At this temperature, the tensile strength is approximately 1303 MPa, the yield strength is 1114 MPa, and the impact energy reaches 80 J, achieving a good balance between strength and toughness.

It should be pointed out that this study mainly explored the influence of tempering temperature on microstructure and quasi-static mechanical properties (tensile and impact) at room temperature, and did not evaluate the material stability under long-term service conditions and fatigue properties under cyclic loading. These are the key evaluation indexes for the engineering application of steel for pressure vessels. In addition, there is no systematic evaluation of residual stress in this study, which is inevitable in practical large-scale engineering applications and may have a significant impact on fatigue life and crack propagation behavior. Therefore, future work should focus on the study of the microstructure evolution and fatigue properties of this steel during long-term aging at high temperature and cyclic loading, and deeply evaluate the influence of residual stress to support its industrial applicability more comprehensively.

## Figures and Tables

**Table 1:** Chemical composition of the experimental steel (wt.%).

Element	C	Mn	Cr	Ni	Mo	V	Al
Experimental	0.24	0.65	1.37	5.04	0.52	0.21	≤1.5

**Table 2:** Mass fractions of constituent elements within the MC phase (wt.%).

Temperature, °C	Cr	Mo	V	C	Σ
425	0	0	0.017	0.004	0.021
475	0.018	0.031	0.028	0.015	0.092
525	0.035	0.076	0.050	0.029	0.190
575	0.058	0.123	0.135	0.061	0.377
625	0.066	0.135	0.146	0.067	0.414

**Table 3:** Mass fractions of constituent elements within the M_3_C phase (wt.%).

Temperature, °C	Fe	Mn	Cr	Mo	Ni	V	C	Σ
425	0.371	0.008	0.015	0.082	0.066	0	0.036	0.493
475	1.355	0.015	0.035	0.045	0.074	0	0.108	1.419
525	1.737	0.028	0.074	0.038	0.087	0.005	0.140	1.837
575	1.728	0.034	0.120	0.099	0.059	0.012	0.145	1.436
625	1.710	0.078	0.211	0.105	0.069	0.015	0.155	2.032

**Table 4:** Chemical formula and density of the MC phase at different tempering temperatures.

Temperature, °C	Chemical Formula	Density (g/cm^3^)
425	VC	5.36
475	(Cr_0.28_Mo_0.27_V_0.45_) C	6.45
525	(Cr_0.28_Mo_0.32_V_0.40_) C	6.61
575	(Cr_0.22_Mo_0.25_V_0.53_) C	6.34
625	(Cr_0.23_Mo_0.25_V_0.52_) C	6.35

**Table 5:** Contribution of each strengthening mechanism to the yield strength, MPa.

Temperature, °C	σ_0_	σ_ss_	σ_g_	σ_d_	σ_p_	Model	Experiment
425	40	603	315	367	281	1202	1140
475	40	490	315	325	482	1191	1136
525	40	430	315	322	505	1146	1114
575	40	256	315	301	615	1049	1103
625	40	126	315	207	670	935	959

## Data Availability

The original contributions presented in this study are included in the article. Further inquiries can be directed to the corresponding author.

## References

[1] Jiao Z.B., Luan J.H., Guo W., Poplawsky J.D., Liu C.T. (2016). Effects of Welding and Post-Weld Heat Treatments on Nanoscale Precipitation and Mechanical Properties of an Ultra-High Strength Steel Hardened by NiAl and Cu Nanoparticles. Acta Mater..

[2] He B.B., Hu B., Yen H.W., Cheng G.J., Wang Z.K., Luo H.W., Huang M.X. (2017). High Dislocation Density-Induced Large Ductility in Deformed and Partitioned Steels. Science.

[3] Kong J., Zhen L., Guo B., Li P., Wang A., Xie C. (2004). Influence of Mo Content on Microstructure and Mechanical Properties of High Strength Pipeline Steel. Mater. Des..

[4] Wei Y., Li Y., Zhu L., Liu Y., Lei X., Wang G., Wu Y., Mi Z., Liu J., Wang H. (2014). Evading the Strength-Ductility Trade-Off Dilemma in Steel through Gradient Hierarchical Nanotwins. Nat. Commun..

[5] Zhang J., Ding H., Misra R.D.K., Wang C. (2015). Microstructural Evolution and Consequent Strengthening through Niobium-Microalloying in a Low Carbon Quenched and Partitioned Steel. Mater. Sci. Eng. A.

[6] Zhao C., Zhang X., Liang X., Song G., Wang B., Guo L., Zhang P., Zhang S. (2025). Effect of Tempering Temperature On Microstructure and Mechanical Properties of Cr-Ni-Mo-V Rotor Steel. Materials.

[7] Tomita Y. (1991). Low-Temperature Improvement of Mechanical Properties of Aisi 4340 Steel through High-Temperature Thermomechanical Treatment. Metall. Trans. A Phys. Metall. Mater. Sci..

[8] Xia B., Zhang P., Wang B., Li X., Zhang Z. (2023). Effects of Quenching Temperature on the Microstructure and Impact Toughness of 50Crmnsivnb Spring Steel. Mater. Sci. Eng. A.

[9] Du Y., Zhou X., Bai R., Zhang Y. (2025). Effect of Intercritical Quenching Temperature on Microstructure and Mechanical Performance of Cr-Ni-Mo-V Steel with Banded Structure. Materials.

[10] Sun G., Wang Q., Li Z. (2024). Microstructural Evolution of High-Nickel Steel During Quenching, Lamellarizing, and Tempering Heat Treatment. J. Mater. Res. Technol..

[11] Zhao L., Wang D., Pang Q., Li W., Du L. (2024). Effect of Quenching-Super Intercritical Quenching-Tempering Process on the Evolution of Microstructure and the Yield Ratio of Ni-Cr-Mo Steel. J. Mater. Res. Technol..

[12] Imanian Ghazanlou S., Mobasher Amini A., Carrier F., Sarkar D.K., Rehman K., Javidani M. (2024). Study of the Microstructure and Mechanical Properties of Steel Grades for Ship Hull Construction. Materials.

[13] Zhou H., Sun X., Tong Z., Cheng G., Xu B., Xiao X., Wang Q., Ran M., Ding H., Zheng W. (2024). Effects of Tempering Temperature On the Precipitation Behaviors of Nanoparticles and their Influences On the Susceptibility to Hydrogen Embrittlement of a Cr-Mo-V Steel. Int. J. Hydrogen Energy.

[14] Byun T.S., Collins D.A., Lach T.G., Choi J.P., Maloy S.A. (2024). Thermomechanical Processing for Improved Mechanical Properties of HT9 Steels. Materials.

[15] Wang Z., Hui W., Chen Z., Zhang Y., Zhao X. (2020). Effect of Vanadium on Microstructure and Mechanical Properties of Bainitic Forging Steel. Mater. Sci. Eng. A.

[16] Yan Z., Liu K., Eckert J. (2020). Effect of Tempering and Deep Cryogenic Treatment on Microstructure and Mechanical Properties of Cr-Mo-V-Ni Steel. Mater. Sci. Eng. A.

[17] Liang X., Fu H., Cui M., Liu G. (2022). Effect of Intercritical Tempering Temperature on Microstructure Evolution and Mechanical Properties of High Strength and Toughness Medium Manganese Steel. Materials.

[18] Liang L., Song G., Wang W., Liang C., He H., Peng Y., Zhao Y., Peng J., Zeng J. (2026). Achieving High Strength and Toughness of a Novel Cost-Saving Cr-Mo-Nb Alloyed Oil Casing Steel Via Quenching and Tempering Process. Mater. Sci. Eng. A.

[19] Saastamoinen A., Kaijalainen A., Heikkala J., Porter D., Suikkanen P. (2018). The Effect of Tempering Temperature on Microstructure, Mechanical Properties and Bendability of Direct-Quenched Low-Alloy Strip Steel. Mater. Sci. Eng. A.

[20] Huang X., Wang L., Wang Z., Wang Z., Liu Q. (2021). Effect of Temperature on Microstructure and Mechanical Properties of Fe-9Ni-2Cu Steel During the Tempering Process. Materials.

[21] Bae K., Moon H., Park Y., Jo I., Lee J. (2022). Influence of Tempering Temperature and Time on Microstructure and Mechanical Properties of Additively Manufactured H13 Tool Steel. Materials.

[22] Zheng Y., Chu S., Zhang L., Wang Q., Qiu G., Zhu L., Guo Z., Xie T., Zhao H., Lu S. (2025). Research on Microstructure Evolution and Carbide Transformation Behavior During the Quenching and Tempering Processes of Secondary Hardening Steel. J. Mater. Res. Technol..

[23] Luo B., Mao W., Zhao Y., Zhao L., Gao H., Ma W., Sheng S., Park N., Bhattacharjee P.P., Wang Q. (2025). Revisiting Rapid Tempering of Martensitic Steel: The Key Role of Tempering Time Over Heating Rate in Carbide Refinement and Precipitation Strengthening. J. Mater. Res. Technol..

[24] Moon J., Choi J., Han S., Huh S., Kim S., Lee C., Lee T. (2016). Influence of Precipitation Behavior on Mechanical Properties and Hydrogen Induced Cracking During Tempering of Hot-Rolled API Steel for Tubing. Mater. Sci. Eng. A.

[25] Sheng J., Deng Y., Cao X., Wang Y., Hu C., Dong H. (2025). Evolution Mechanism of Multi-Precipitation Regulates Mechanical Properties and High-Temperature Strength in Medium-Alloy Structural Steel. Materials.

[26] Jiang J., Liu Y., Liu C. (2022). Effect of Tempering Temperature On the Microstructure, Mechanical Properties and Abrasive Wear Behavior of a New C-Cr-Mo-V Alloy Steel Used in Tbm Cutter Ring. J. Mater. Res. Technol..

[27] Wen T., Hu X., Song Y., Yan D., Rong L. (2013). Carbides and Mechanical Properties in a Fe–Cr–Ni–Mo High-Strength Steel with Different V Contents. Mater. Sci. Eng. A.

[28] Zheng K., Wang H., Yu F., Lin S., Zhong Z., Wang C., Liang J., Cao W. (2026). The Effect of Tempering Temperature on the Microstructure and Properties of a Novel High-Temperature Bearing Steel. Materials.

[29] Dong J., Zhou X., Liu Y., Li C., Liu C., Guo Q. (2017). Carbide Precipitation in Nb-V-Ti Microalloyed Ultra-High Strength Steel During Tempering. Mater. Sci. Eng. A.

[30] Shi K., Zhao F., Liu Y., Yin S., Yang R. (2022). The Effect of the Pre-Existing VC On the Evolution of Precipitate and Mechanical Properties in the H13 Steel. Materials.

[31] Chen J., Jin P., Wang S., Zhu C., Xu M., Jia Z., Liu X., Zhao C., Zhang C., Huang J. (2025). A Novel Ni-Mo-W-V Martensitic Steel for Hot Working Dies: Improved Elevated-Temperature Mechanical Properties and Wear Resistance Via Thermally Stable Mc Nanoprecipitates. Tribol. Int..

[32] (2021). Metallic Materials—Tensile Testing—Part 1: Method of Test at Room Temperature.

[33] (2020). Metallic Materials—Charpy Pendulum Impact Test Method.

[34] (2004). Nanometer Powder—Determination of Particle Size Distribution—Small Angle X-Ray Scattering Method.

[35] HajyAkbary F., Sietsma J., Böttger A.J., Santofimia M.J. (2015). An Improved X-Ray Diffraction Analysis Method to Characterize Dislocation Density in Lath Martensitic Structures. Mater. Sci. Eng. A.

[36] Soleimani M., Mirzadeh H., Dehghanian C. (2020). Effects of Tempering on the Mechanical and Corrosion Properties of Dual Phase Steel. Mater. Today Commun..

[37] Ji Y.P., Liu Z.C., Ren H.P. (2011). Morphology and Formation Mechanism of Martensite in Steels with Different Carbon Content. Adv. Mater. Res..

[38] Sun C., Fu P., Liu H., Liu H., Du N. (2018). Effect of Tempering Temperature on the Low Temperature Impact Toughness of 42Crmo4-V Steel. Metals.

[39] Liu M., Wang C.H., Dai Y.C., Li X., Cao G.H., Russell A.M., Liu Y.H., Dong X.M., Zhang Z.H. (2017). Effect of Quenching and Tempering Process on Sulfide Stress Cracking Susceptibility in Api-5Ct-C110 Casing Steel. Mater. Sci. Eng. A.

[40] Zhao D., Zhang S., Zhang H., Li S., Xiao H., Wang Y., Wang X. (2019). Effects of Tempering Temperature on the Microstructure and Mechanical Properties of T92 Heat-Resistant Steel. Metals.

[41] Liu H., Fu P., Liu H., Sun C., Ma X., Li D. (2018). Microstructure Evolution and Mechanical Properties in 718H Pre-Hardened Mold Steel During Tempering. Mater. Sci. Eng. A.

[42] Sánchez Egea A.J., González Rojas H.A., Celentano D.J., Jorba Perió J., Cao J. (2017). Thermomechanical Analysis of an Electrically Assisted Wire Drawing Process. J. Manuf. Sci. Eng..

[43] Wang H., Feng H., Li H., Zhang S., Zhu H., Jiang Z. (2024). Effect of Isothermal Tempering Time and Temperature on Microstructure and Hardness of 3Cr5Mo2Sivn Hot-Work Die Steel. Mater. Charact..

[44] Zhang C., Wang Q., Ren J., Li R., Wang M., Zhang F., Yan Z. (2012). Effect of Microstructure on the Strength of 25Crmo48V Martensitic Steel Tempered at Different Temperature and Time. Mater. Des..

[45] Yan P., Liu Z., Bao H., Weng Y., Liu W. (2014). Effect of Normalizing Temperature On the Strength of 9Cr-3W-3Co Martensitic Heat Resistant Steel. Mater. Sci. Eng. A.

[46] Maropoulos S., Paul J.D.H., Ridley N. (2013). Microstructure-Property Relationships in Tempered Low Alloy Cr-Mo-3·5Ni-V Steel. Mater. Sci. Technol..

[47] Yu W., Jin R., Han L., Xie D., Sun T., Wang M., Shi J. (2025). Effect of Ti and Tinb Microalloying On Microstructures and Mechanical Properties of 2200 Mpa Low-Alloy Ultra-High-Strength Steels. Metals.

[48] Chang L.C., Bhadeshia H.K.D.H. (1994). Carbon Content of Austenite in Isothermally Transformed 300M Steel. Mater. Sci. Eng. A.

[49] Geng H., Sun X., Guo X., Zhao Y., Yin X., Du Z. (2024). Achieving 1.7 Gpa Considerable Ductility High-Strength Low-Alloy Steel Using Hot-Rolling and Tempering Processes. Materials.

[50] Wang Y., Sun J., Jiang T., Sun Y., Guo S., Liu Y. (2018). A Low-Alloy High-Carbon Martensite Steel with 2.6 Gpa Tensile Strength and Good Ductility. Acta Mater..

[51] Liu T., Cao Z., Wang H., Wu G., Jin J., Cao W. (2020). A New 2.4 Gpa Extra-High Strength Steel with Good Ductility and High Toughness Designed by Synergistic Strengthening of Nano-Particles and High-Density Dislocations. Scr. Mater..

[52] Zhang K., Li Z., Wang Z., Sun X., Yong Q. (2016). Precipitation Behavior and Mechanical Properties of Hot-Rolled High Strength Ti–Mo-Bearing Ferritic Sheet Steel: The Great Potential of Nanometer-Sized (Ti, Mo)C Carbide. J. Mater. Res..

[53] Claesson E., Magnusson H., Hedström P. (2023). Scanning Precession Electron Diffraction Study of Carbide Precipitation Sequence in Low Alloy Martensitic Cr-Mo-V Tool Steel. Mater. Charact..

[54] Yang C., Ji Y., Xu T., Hu C., Dong H. (2025). Effect of Cyclic Quenching On Martensitic Variant Selection, Mechanical Behavior and Toughening Mechanisms in Medium Carbon Cr-Ni-Mo-V Steel. Mater. Sci. Eng. A.

[55] Krauss G. (2017). Tempering of Lath Martensite in Low and Medium Carbon Steels: Assessment and Challenges. Steel Res. Int..

[56] Lee C., Seol W., Kim B.H., Kim S., Jang J.H., Lee T., Moon S., Kim S. (2019). Investigation on New Type of Fracture in Cr-Mo-V Steel Slab. Eng. Fail. Anal..

[57] Ren Q., Kou Z., Wu J., Hou T., Xu P. (2023). Effect of Tempering Temperature on Microstructure and Mechanical Properties of 35Crmo Steel. Metals.

[58] Luo Y., Peng J., Wang H., Wu X. (2010). Effect of Tempering on Microstructure and Mechanical Properties of a Non-Quenched Bainitic Steel. Mater. Sci. Eng. A.

[59] Liu J., Yu H., Zhou T., Song C., Zhang K. (2014). Effect of Double Quenching and Tempering Heat Treatment on the Microstructure and Mechanical Properties of a Novel 5Cr Steel Processed by Electro-Slag Casting. Mater. Sci. Eng. A.

